# Supplementary material for: A Ubiquitously Expressed UDP-Glucosyltransferase, UGT74J1, Controls Basal Salicylic Acid Levels in Rice
Source: Plants (Basel). 2021 Sep 10;10(9):1875. doi: 10.3390/plants10091875 (PMC8469147; doi:10.3390/plants10091875)
Supplement: Supplementary file 1 [file plants-10-01875-s001.zip › plants-1332855-supplementary.pdf]

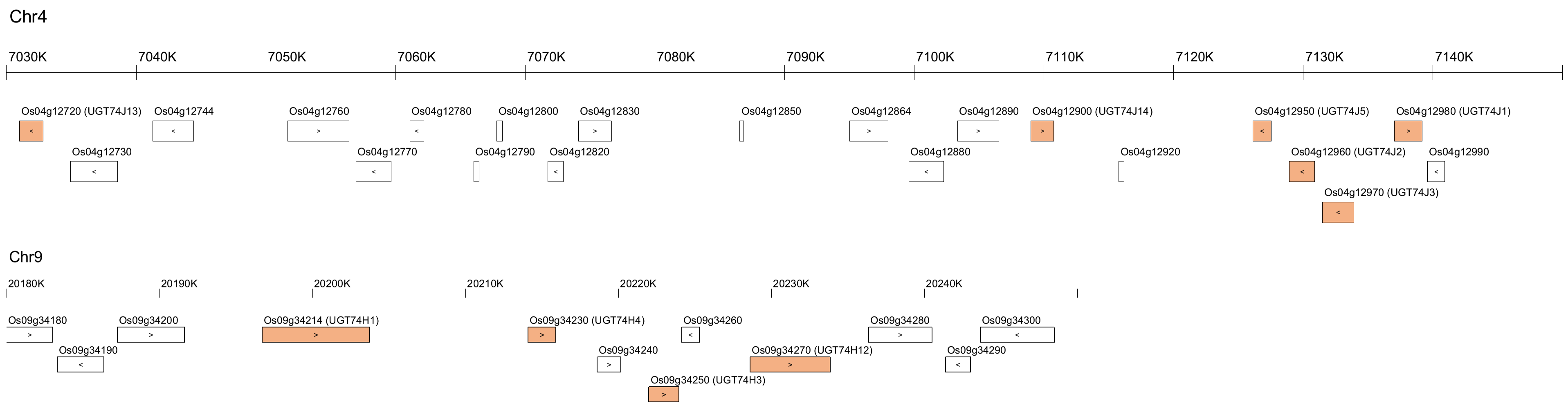

Figure S1. Schematic model of the UGT gene clusters on chromosomes 4 and 9.
The UGT genes are indicated by orange boxes. The locus name and chromosomal position of each gene conform to the MSU Rice Genome Annotation Project Database.

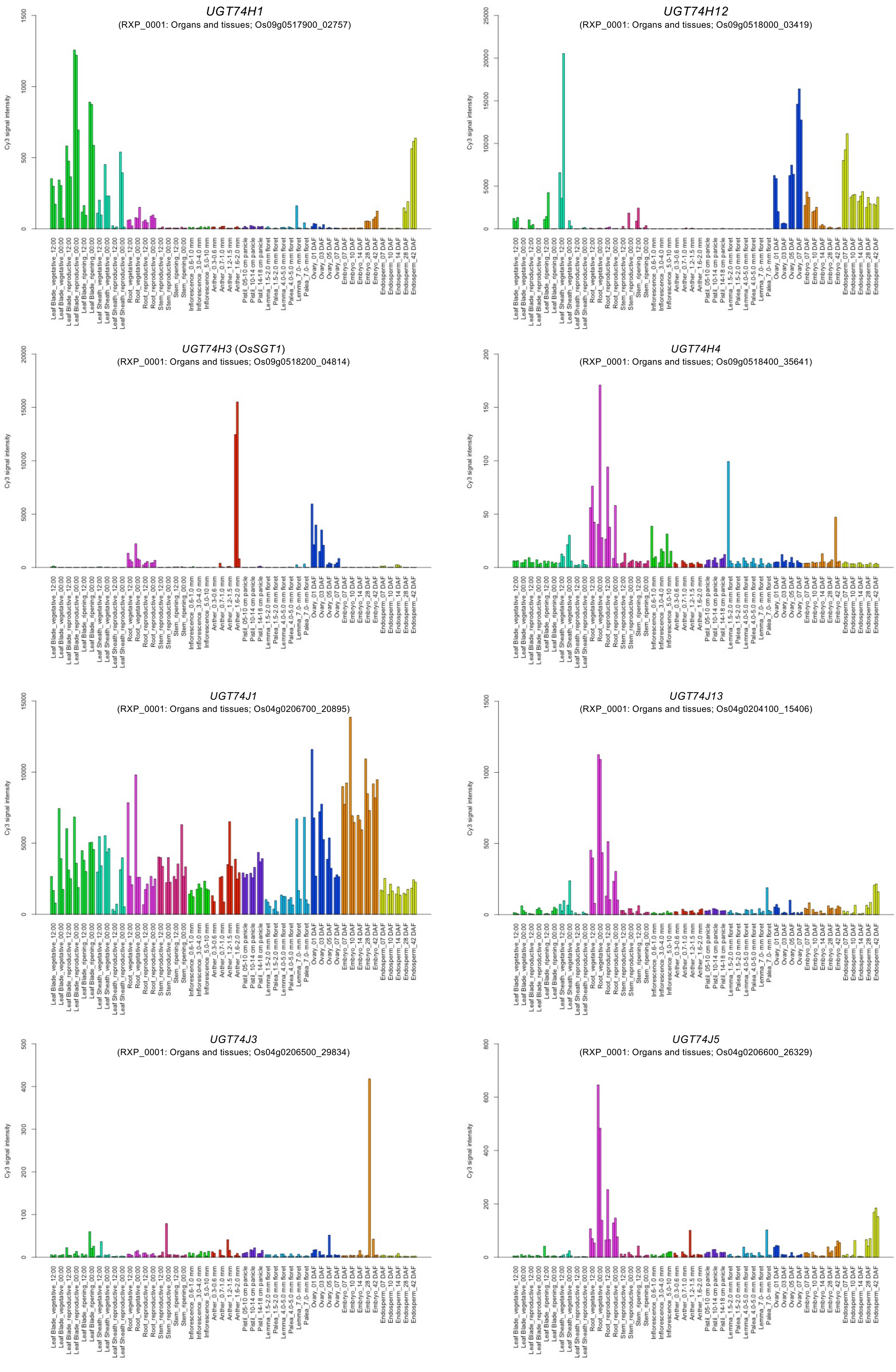

Figure S2. Tissue specific expression of the *UGT74* genes.
(A) Raw signal intensity graphs from a dataset (ID: RXP_0001) registered in RicexPro microarray database (https://ricexpro.dna.affrc.go.jp/).

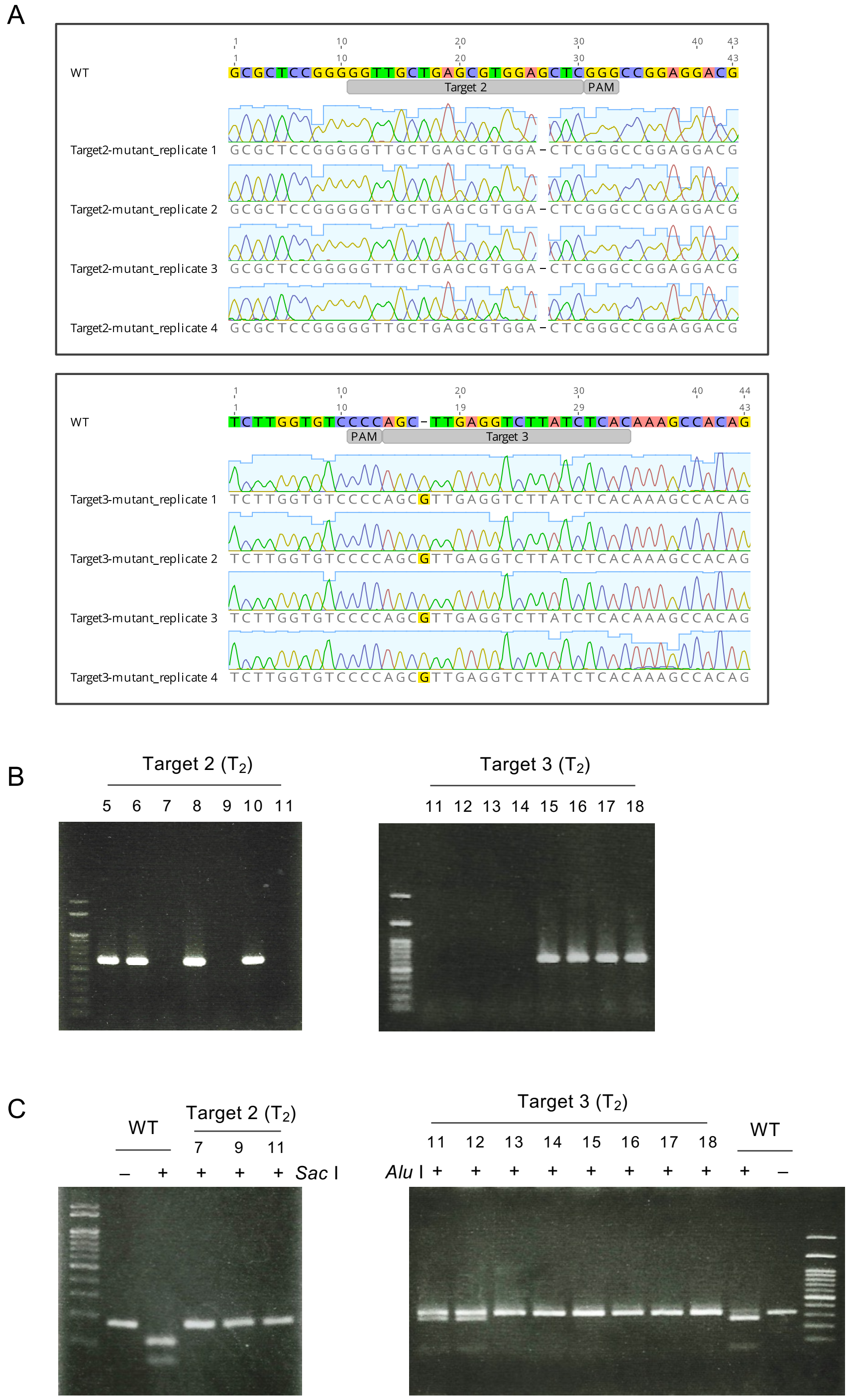

Figure S3. Screening for the *ugt74j1* mutants.
(A) Partial sequence of *UGT74J1* in the mutants. Genomic DNA was extracted from two independent T_1 plants. The *UGT74J1* fragments were amplified and cloned. Sequences from four independent clones are aligned with the wild type. (B) Segregation of the CRISPR/Cas9 expression cassette in T_2 transgenic rice. *Hygromycin phosphotransferase* partial fragments were amplified. (C) Segregation of the *UGT74J1* mutation in T_2 transgenic rice. The genotype of each plant was observed by a CAPS assay.

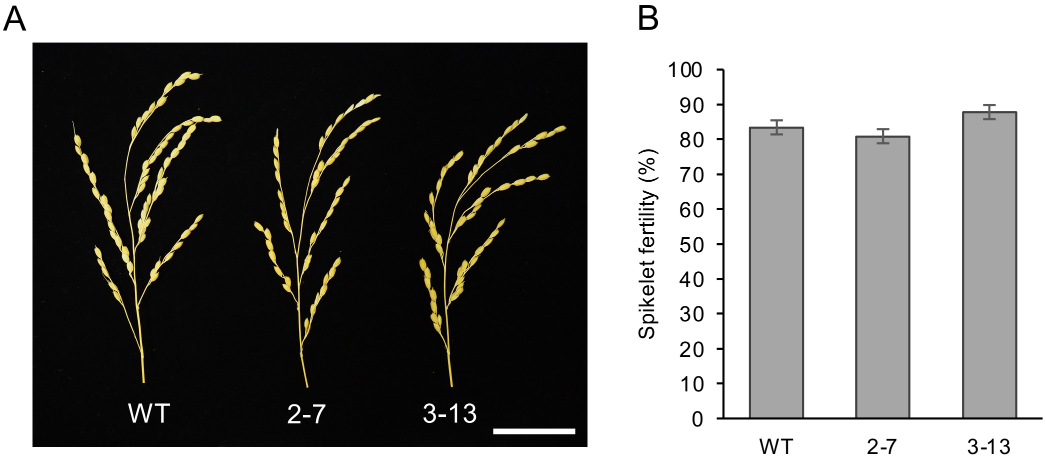

Figure S4. Spikelet fertility of the *ugt74j1* mutants.
(A) A photo of mature panicles of the *ugt74j1* mutants and wild type. Scale bar represents 5 cm. (B) Spikelet fertility of the *ugt74j1* mutants and wild type. The data are represented as the mean ± SE (n = 6). Fertility rates of the mutants was not significantly different from that of the wild type (*t*-test, $p > 0.05$).

Table S1. Screening for defense genes using microarray.

MSU ID	RAP ID	Description	Gene name	Fold change (2-7/ wild type)
LOC_Os03g18850	Os03g0300400	Pathogen-related protein (JIOsPR10).	JIOsPR10	18.96
LOC_Os11g37960	Os11g0592100	Barwin.	OsPR4b	16.57
LOC_Os01g47070	Os01g0660200	Acidic class III chitinase OsChib3a precursor (Chitinase) (EC 3.2.1.14).	OsChib3a	11.49
LOC_Os01g28450	Os01g0382000	Pathogenesis-related protein 1 precursor (PR-1).	OsPR1#011	10.30
LOC_Os01g51570	Os01g0713200	Beta-1,3-glucanase precursor.	Gns10	10.20

Table S2. Accession numbers of UDP-glucosyltransferases used in phylogenetic analysis

**Name**	**Species**	**Gene ID**	**Protein ID**
UGT74B1	*A. thaliana*	At1g24100	NP_173820.1
UGT74C1	*A. thaliana*	At2g31790	NP_180738.1
UGT74D1	*A. thaliana*	At2g31750	NP_180734.1
UGT74E2	*A. thaliana*	At1g05680	NP_172059.1
UGT74F1	*A. thaliana*	At2g43840	NP_973682.1
UGT74F2	*A. thaliana*	At2g43820	NP_181910.1
UGT75B1	*A. thaliana*	At1g05560	NP_563742.1
UGT75B2	*A. thaliana*	At1g05530	NP_172044.1
UGT75C1	*A. thaliana*	At4g14090	NP_193146.1
UGT84A1	*A. thaliana*	At4g15480	NP_193283.2
UGT84A2	*A. thaliana*	At3g21560	NP_188793.1
UGT84A3	*A. thaliana*	At4g15490	NP_193284.1
UGT84A4	*A. thaliana*	At4g15500	NP_193285.1
UGT84B1	*A. thaliana*	At2g23260	NP_179907.1
UGT84B2	*A. thaliana*	At2g23250	NP_179906.1
UGT74A2	*O. sativa*	LOC_Os03g48740	LOC_Os03g48740.1
UGT74J1	*O. sativa*	LOC_Os04g12980	LOC_Os04g12980.1
UGT74J13	*O. sativa*	LOC_Os04g12720	LOC_Os04g12720.1
UGT74J14	*O. sativa*	LOC_Os04g12900	LOC_Os04g12900.1
UGT74J2	*O. sativa*	LOC_Os04g12960	LOC_Os04g12960.1
UGT74J3	*O. sativa*	LOC_Os04g12970	LOC_Os04g12970.1
UGT74J5	*O. sativa*	LOC_Os04g12950	LOC_Os04g12950.1
UGT74H1	*O. sativa*	LOC_Os09g34214	LOC_Os09g34214.1
UGT74H12	*O. sativa*	LOC_Os09g34270	LOC_Os09g34270.1
UGT74H3_OsSGT1	*O. sativa*	LOC_Os09g34250	LOC_Os09g34250.1
UGT74H4	*O. sativa*	LOC_Os09g34230	LOC_Os09g34230.1
UGT75E1	*O. sativa*	LOC_Os11g04860	LOC_Os11g04860.1
UGT75F2	*O. sativa*	LOC_Os11g25990	LOC_Os11g25990.1
UGT75G1	*O. sativa*	LOC_Os06g39270	LOC_Os06g39270.1
UGT75H1	*O. sativa*	LOC_Os06g39330	LOC_Os06g39330.1
UGT75J1	*O. sativa*	LOC_Os02g10880	LOC_Os02g10880.1
UGT75K1	*O. sativa*	LOC_Os01g08440	LOC_Os01g08440.1
UGT75K2	*O. sativa*	LOC_Os05g08750	LOC_Os05g08750.1
UGT84C1	*O. sativa*	LOC_Os02g09510	LOC_Os02g09510.1
UGT84D1	*O. sativa*	LOC_Os05g47950	LOC_Os05g47950.1
UGT84E1	*O. sativa*	LOC_Os01g49230	LOC_Os01g49230.1
UGT84E2	*O. sativa*	LOC_Os01g49240	LOC_Os01g49240.1

Table S3. Primers used in this study

**Gene name**	**Experiment**	**RAP ID**	**MSU ID**	**Forward primer sequense (5' to 3')**	**Reverse primer sequense (5' to 3')**
OsACT1	Semi-quantitative RT-PCR	Os03g0718100	LOC_Os03g50890	TCCATCTTGGCATCTCTCAG	GTACCCTCATCAGGCATCTG
UGT74H1	Semi-quantitative RT-PCR	Os09g0517900	LOC_Os09g34214	GGATCGAGGAGGTGATGCG	TGCAGAGAATTTCCCCCACC
UGT74H12	Semi-quantitative RT-PCR	Os09g0518000	LOC_Os09g34230	ATTTCGCGTGCAATAACCGG	GAACTCGTCCTTCCTAGCGG
UGT74H3_OsSGT1	Semi-quantitative RT-PCR	Os09g0518200	LOC_Os09g34250	GGTGTGTGAGGGAGGTGATG	CATCCGACTGTGCCCATTTC
UGT74H4	Semi-quantitative RT-PCR	Os09g0518400	LOC_Os09g34270	GAGGAGGTGGAGAGGAAGGT	TTCCAAATTCATGGCGAGGC
UGT74J1	Semi-quantitative RT-PCR	Os04g0206700	LOC_Os04g12980	GAGTTTGCCTCGAAGTACGC	CTTGTCCAAATATCGTTGAGCA
UGT74J13	Semi-quantitative RT-PCR	Os04g0204100	LOC_Os04g12720	ACAGGAGGAATGCTGCAAGG	TCAGCACCTTGACCTCCATG
UGT74J14	Semi-quantitative RT-PCR	Os04g0206000	LOC_Os04g12900	TGTTAGCGATGCCTCAGTGG	ACTTGATTAGTCATCATCTGGCCA
UGT74J2	Semi-quantitative RT-PCR	–	LOC_Os04g12950	GCTCTGTCACGAAGGAGGAG	AACTCCATCTTGTCGTCTGCTA
UGT74J3	Semi-quantitative RT-PCR	Os04g0206500	LOC_Os04g12960	TGGTGACAGGAAGGAGGACT	CAGCACCTTGACCTCCATGA
UGT74J5	Semi-quantitative RT-PCR	Os04g0206600	LOC_Os04g12970	AACTCGACGTTGGAGGCAAT	TCCCGAATGCACCTCTCAAC
OsACT1	Real-time PCR	Os03g0718100	LOC_Os03g50890	TCTCTCTGTATGCCAGTGGTCG	GTCGAGACGAAGGATAGCATGG
OsPR1#011	Real-time PCR	Os01g0382000	LOC_Os01g28450	ACGGGCGTACGTACTGGCTA	CTCGGTATGGACCGTGAAG
Gns10	Real-time PCR	Os01g0713200	LOC_Os01g51570	CGACGAGAACGGCAAGCCTG	TGGCCTATCACGGGAGCAAC
OsPR4b	Real-time PCR	Os11g0592100	LOC_Os11g37960	CGTCTTCTCCAAGATCGACA	CGAACTGGTAGTCGACGATG
OsChib3a	Real-time PCR	Os01g0660200	LOC_Os01g47070	TCTACGACGTGCAGAACAACTTCAG	TCCAACTCAACCACTGTGCAAGTAA
JIOsPR10	Real-time PCR	Os03g0300400	LOC_Os03g18850	CCTCAGCCATGCCATTCAG	CTTGTCCACGTCCAGGAACTC
UGT74J1_Target 2	Mutant screening	Os04g0206700	LOC_Os04g12980	GTTCTTCTCGCAGCCGTG	AGGAACGGCGGGTACGACTC
UGT74J1_Target 3	Mutant screening	Os04g0206700	LOC_Os04g12980	GAACTCCGTGACAAGTGC	CTGTAATCGTAGCCTTCTGAC
Hygromycin phosphotransferase	Mutant screening	–	–	GGCGACCTCGTATTGGGAATCCCCGAACAT	CGGAGGGCGAAGAATCTCGTGCTTTCAGCT
